# Factors associated with favorable survival outcomes for Asians with hepatocellular carcinoma: A sequential matching cohort study

**DOI:** 10.1371/journal.pone.0214721

**Published:** 2019-04-03

**Authors:** Zhensheng Wang, Xiangjun Gu, Aaron P. Thrift

**Affiliations:** 1 Section of Epidemiology and Population Sciences, Department of Medicine, Baylor College of Medicine, Houston, Texas, United States of America; 2 Dan L Duncan Comprehensive Cancer Center, Baylor College of Medicine, Houston, Texas, United States of America; Centre de Recherche en Cancerologie de Lyon, FRANCE

## Abstract

**Background:**

Overall 5-year survival rates for patients diagnosed with hepatocellular carcinoma (HCC) are poor, but vary by race/ethnicity. We undertook a comprehensive assessment of underlying contributing factors to the favorable survival outcomes of HCC among Asians compared with non-Hispanic whites (NHW).

**Methods:**

We identified 1,284 Asian and 7,072 NHW patients newly diagnosed with HCC between 1994 and 2011 in the Surveillance, Epidemiology and End Results (SEER)-Medicare linked database. We used a novel three-step sequential matching approach to identify demographic, presentation and treatment factors that may explain survival differences between Asians and NHWs. Hazard ratios (HRs) and corresponding 95% confidence intervals (CIs) for the association between Asian race and risk of HCC-related mortality were estimated using Cox proportional hazards models.

**Results:**

The absolute difference in 5-year survival rates between Asians and NHWs was 8.4% (95% CI: 4.6%-12.0%) in the demographics match analysis. The disparity remained unchanged after additionally matching on stage, grade and comorbidities in the presentation match analysis. However, in the treatment match analysis, which accounts for differences in demographic, presentation and treatment factors, the absolute difference in 5-year survival rates was reduced to 5.8% (95% CI: 2.6%-9.3%). Treatment differences explained more of survival disparity in Asian and NHW patients with localized disease than for those with regional or distant stage HCC.

**Conclusions:**

Asian patients with HCC continue to have more favorable survival outcomes than NHWs with HCC. This persistent disparity seems to be more related to treatment differences than to differences in presentation characteristics including stage.

## Introduction

Hepatocellular carcinoma (HCC) is the most common type of liver cancer, accounting for around 75% of an estimated 42,220 incident liver cancer cases in the United States during 2018 [1]. The incidence of HCC has increased dramatically in the US [2]. Moreover, the mortality rate for liver cancer has increased by more than 40% for both men and women since 2000 [3]. Among patients with HCC, there is compelling evidence for a persistent racial disparity in survival after diagnosis [4–7]. Specifically, Asians have better overall observed 5-year survival rates compared with other races/ethnicities [7–10].

The factors underlying the better survival outcomes of Asians with HCC compared with non-Hispanic whites (NHWs) with HCC are likely multifactorial, including differences in stage at diagnosis, access to screening, existing comorbid conditions at diagnosis, socioeconomic status and receipt of treatment. Tumor stage at diagnosis is one of the strongest prognostic factors for HCC; the 5-year survival rate is 31.3% for localized stage HCC but only 2.4% for distant stage [11]. A previous study reported that Asians with HCC are 13% less likely than NHWs to be diagnosed at advanced stage [12]. Another potentially important contributing factor to the survival disparity is differences in receipt of surgical therapy including liver transplantation and hepatectomy. While Asians are less likely to receive liver transplantation [13,14], they are more likely than NHWs with HCC to receive hepatectomy [12] and have been shown to respond better (in terms of longer survival time) from the procedure than NHWs [9]. Socioeconomic factors, including marital status, have also been shown to be explain some of the survival differences between Asians and NHWs with HCC [10]. For example, keeping in marriage has been shown to be associated with a higher likelihood of receiving surgical treatment and subsequent favorable outcomes for multiple cancer sites including liver cancer [15,16].

The proportion of HCCs that are attributable to hepatitis B virus (HBV) infection, hepatitis C virus (HCV) infection, alcoholic liver disease (ALD) or non-alcoholic fatty liver disease (NAFLD) differs by race/ethnicity. As there is evidence that HCC survival rates may differ by etiology (e.g., patients with HBV-related HCC has been shown to have better 5-year survival rates than patients with HCV-related HCC, independent of tumor stage [17]), this may also contribute to the observed racial disparities in HCC survival.

Although previous studies have examined the extent of the racial disparity in HCC survival, the main purpose of this study was to understand the nature of the disparity. Here, we asked whether (i) Asian patients who present like NHW patients are subsequently treated as NHW patients are treated, and (ii) if not, to what extent a disparity in receipt of treatment explains the observed survival disparity. We assessed the magnitude of the disparity and determined the relative contributions of presentation factors (and treatment after presentation) to differences in survival experienced by Asian and NHW patients.

## Materials and methods

### Study population

We identified all patients diagnosed with a first primary HCC during 1994 and 2011 with self-reported race as Asian/Pacific Islander or NHW from 17 cancer registries in the National Cancer Institute’s (NCI) Surveillance, Epidemiology and End Results (SEER)-Medicare linked database. In brief, SEER is a cancer surveillance program with published data on cancer incidence and survival covering approximately 34% of the US population [18]. By linking with the Medicare database, detailed information including inpatient, outpatient and physicians medical claims are available among Medicare beneficiaries with cancer [19]. Incident HCC cases were defined by International Classification of Diseases for Oncology, Third Edition (ICD-O-3) primary site code C22.0 and histology codes 8170–8175. Other inclusion criteria were: 1) age above 65 years; 2) continuous Medicare enrollment and no HMO enrollment at least 12 months prior to HCC diagnosis; and 3) HCC cases not identified via death certificates or autopsy. This research was approved by the Institutional Review Board at Baylor College of Medicine (Board 1 for protocol H-39135). A waiver of consent was obtained because the study was a retrospective analysis and all data were de-identified.

### Study variables

The included study variables were classified into three broad categories: demographics, presentation and treatment. Demographic variables included age at diagnosis (in years), year of diagnosis, gender, socioeconomic status (SES; high vs. low), SEER registry site, current married status (yes vs. no), and rurality status (metropolitan vs. urban). Presentation variables included the NCI comorbidity index (categorized as 0, 1, 2 or ≥3), cancer stage at diagnosis (localized, regional, distant or unknown) and tumor differentiation grade (I, II, III, IV or unknown). Treatment variables included surgery status (no surgery, tumor destruction, resection, liver transplantation or unknown), radiation therapy (yes vs. no) and chemotherapy (yes vs. no). Surgery type definitions are listed in **S1 Table**. SES was based on census tract medium income, percentage of persons over 25 years with <12 year education, and percentage of persons living below poverty threshold defined by the US government [20] (**S2 Table**). The total score ranged from 0 to 9 and SES was categorized as low (score: 0–7) or high (score: 8–9). The details of NCI comorbidity index has been described previously [21]. In brief, it is an adaption of the original Charlson comorbidity index and is cancer-specific. To define each of the 16 conditions, ICD-9-CM diagnosis and procedure codes were identified from inpatient, outpatient and physicians’ claim data up to one year prior to HCC diagnosis. A weight from 1 to 6 was assigned to each condition based on its association with one-year mortality. We expanded the definitions of mild liver diseases and moderate to severe liver diseases to include risk factors for HCC such as HBV and HCV infection, NAFLD, and ALD. The definition of each condition and associated weight assignment are listed in **S3 Table**.

### Statistical analysis

Primary outcomes of analysis included 1-, 2- and 5-year observed survival rates from HCC-specific deaths. Patients were followed from HCC diagnosis date until death due to any cause, maximum claim date, or December 31, 2012, whichever occurred first, whereby each patient had the opportunity for a minimum of 1-year of follow-up after their HCC diagnosis date.

We conducted a three-step sequential multivariate matching procedure to individually or frequency match the NHWs to all Asians without replacement (1:1 matching ratio), which has been described previously [22,23]. The matched NHW population sub-cohort (i.e., a subset of the total unmatched NHW cohort) changed according to the criteria at each step of sequential matching. The analysis consisted of the following steps: 1) a demographics match, which matched NHWs to Asians on age at HCC diagnosis (matching by minimizing age difference), year of diagnosis (within 5 years), sex, SES and SEER site; 2) a presentation match, which matched exactly on cancer stage, grade, and category of NCI comorbidity index as well as minimizing a propensity score on demographics variables (details below); and 3) a treatment match, which matched exactly on treatment variables (surgery, chemotherapy, and radiation therapy) and minimizing a propensity score on demographics and presentation variables.

A propensity score is the conditional probability of exposure controlling for all confounding factors. It has been previously shown that by matching on subjects with minimized differences of a propensity score, the exposure effects on the outcome could be investigated while effectively adjusting for confounders [24]. We applied a logistic regression model to regress demographic or presentation factors on race (Asian or NHW). Predicted probabilities for each Asian and NHW patient were obtained after fitting the model and the distances between one Asian patient and all other treatment matched NHW patients by taking the absolute differences of two predicted probabilities. Finally, we take the minimum distance to find the NHW patient who best matches the Asian patient. In order to examine the success of the patient matching process, standardized differences (defined as mean difference between Asians and NHWs as a fraction of the standard deviation [SD] before matching) were compared for each matching variable before and after matching, and we considered a standardized difference less than 0.1 SDs as successfully matched.

For each of the matched cohorts, we compared median survival between Asians and matched NHWs using the non-parametric Wilcoxon sign-rank test, and compared the 1-, 2- and 5-year survival rates using the lifetable method. Paired Cox proportional hazards models were conducted to examine survival over time and to estimate hazards ratios (HRs) and corresponding 95% confidence intervals (CIs). We used the bootstrap method to obtain standard errors for the paired differences in survival [25].

Stratified analysis by stage at diagnosis (localized vs. regional or distant) and calendar year of diagnosis (1994–2006 vs. 2007–2011) were performed to further examine the matching factors and racial disparity in HCC survival on homogeneous subgroups of HCC patients. We chose 2007 as the cutoff year due to main breakthroughs in targeted chemotherapy such as sorafenib (Nexavar) having shown survival advantage in phase III clinical trial [26] and subsequently approved by Food and Drug Administration (FDA) for advanced HCC treatment [27].

All analysis were conducted in SAS 9.4 (SAS Institute, Cary, NC). Two-sided P-values less than 0.05 were considered statistical significant.

## Results and discussion

**Fig 1:**
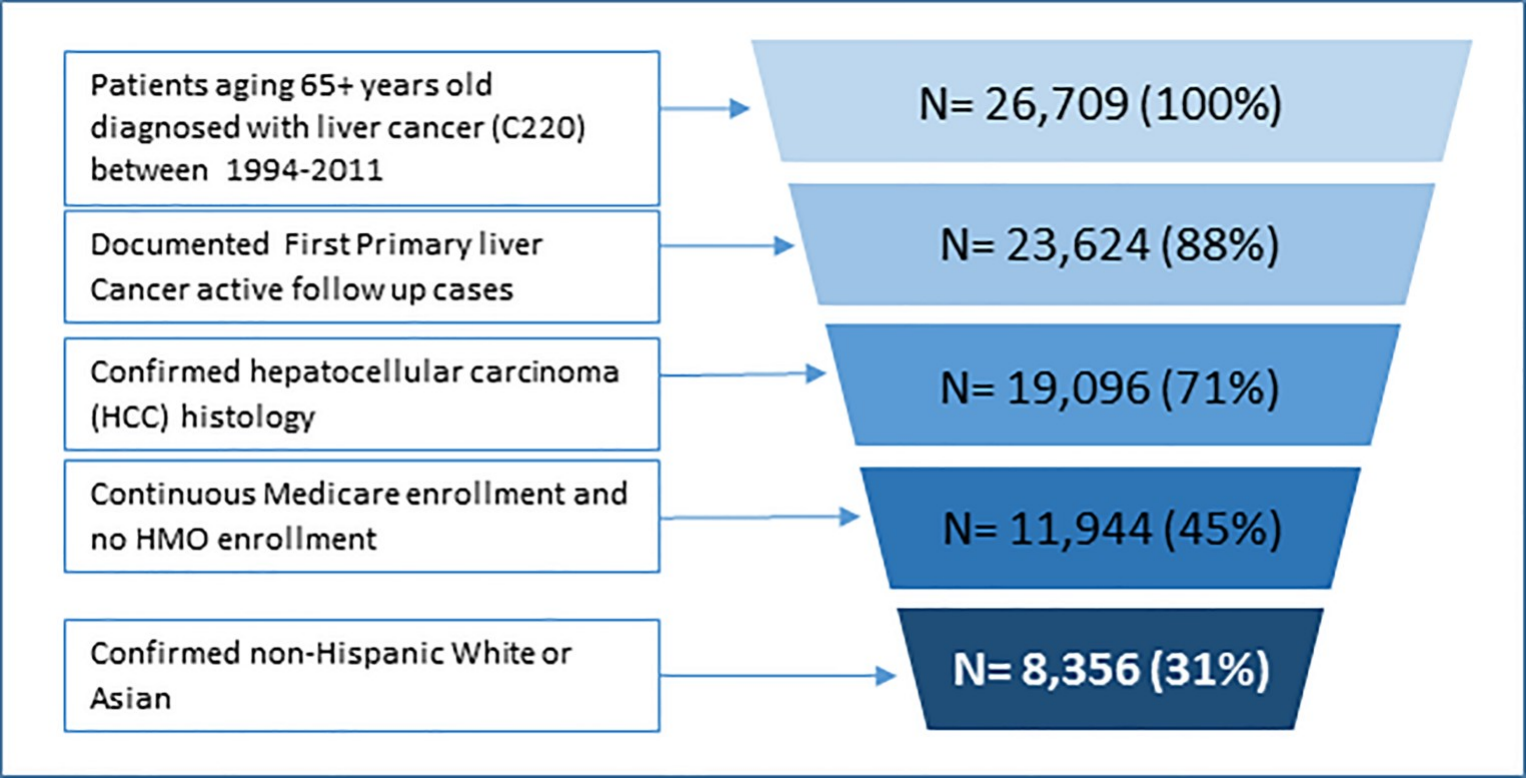
Flow diagram showing included non-Hispanic white and Asian Hepatocellular carcinoma patients from SEER-Medicare datasets.

A total of 8,356 HCC patients (1,284 Asians and 7,072 NHWs) met the study inclusion criteria and were included in the primary analysis (**Fig 1**). Compared with unmatched NHW patients with HCC, Asian patients with HCC were more likely to be female, more likely to receive chemotherapy and surgery (tumor destruction or resection), but less likely to receive radiation therapy (**Table 1**).

**Table 1:** Characteristics of Asian and non-Hispanic white HCC patients by matching criteria.

Variable			Non-Hispanic White Patients, n (%)			
		Asian Patients	Treatment Match	Presentation Match	Demographic Match	All Whites (Unmatched)
		(n = 1284)	(n = 1284)	(n = 1284)	(n = 1284)	(n = 7072)
**Mean age at diagnosis (SD), y**		75.40 (6.19)	75.15 (6.59)	75.45 (6.64)	75.41 (6.39)	75.71 (6.45)
**Mean diagnosis year (SD)**		2004 (4.39)	2004 (4.72)	2004 (4.70)	2004 (4.77)	2004 (4.66)
**Female**		451 (35.12)	443 (34.50)	426 (33.18)	451 (35.12)	**2223 (31.43)**
**SES**						
** **	**High**	>632 (>49.22)	>671 (>52.26)	>646 (>50.31)	>632 (>49.22)	3392 (47.96)
** **	**Low**	641 (49.92)	602 (46.88)	627 (48.83)	641 (49.92)	3667 (51.85)
	**Unknown**	<11 (<0.86)	<11 (<0.86)	<11 (<0.86)	<11 (<0.86)	13 (0.19)
**SEER registry site**						
1	San Francisco	201 (15.65)	206 (16.04)	196 (15.26)	201 (15.65)	**288 (4.07)**
2	Connecticut	16 (1.25)	12 (0.93)	16 (1.25)	16 (1.25)	**635 (8.98)**
20	Detroit	18 (1.40)	14 (1.09)	19 (1.48)	18 (1.40)	**626 (8.85)**
21	Hawaii	39 (3.04)	36 (2.80)	32 (2.49)	39 (3.04)	**39 (0.55)**
22	Iowa	**	**	**	**	**486 (6.87)**
23	New Mexico	**	**	**	**	**140 (1.98)**
25	Seattle	98 (7.63)	106 (8.26)	96 (7.48)	98 (7.63)	**519 (7.34)**
26	Utah	**	**	**	**	**175 (2.47)**
27	Atlanta	13 (1.01)	12 (0.93)	13 (1.01)	13 (1.01)	**197 (2.79)**
31	San Jose	148 (11.53)	126 (9.81)	119 (9.27)	148 (11.53)	**153 (2.16)**
35	Los Angeles	380 (29.60)	344 (26.79)	344 (26.79)	380 (29.60)	**486 (6.87)**
37	Rural Georgia	**	**	**	**	**22 (0.31)**
41	Greater California	303 (23.60)	350 (27.26)	369 (28.74)	303 (23.60)	**984 (13.91)**
42	Kentucky	**	**	**	**	**468 (6.66)**
43	Louisiana	**	12 (0.93)	**	**	**419 (5.92)**
44	New Jersey	37 (2.88)	40 (3.12)	46 (3.58)	37 (2.88)	**930 (13.15)**
47	Greater Georgia	**	**	**	**	**505 (7.14)**
**NCI Comorbidity Index**						
	0	285 (22.20)	331 (25.78)	285 (22.20)	**357 (27.80)**	**1876 (26.53)**
	1	407 (31.70)	382 (29.75)	407 (31.70)	**332 (25.86)**	**1854 (26.22)**
	2	255 (19.85)	231 (17.99)	255 (19.85)	**225 (17.52)**	**1274 (18.01)**
	> = 3	337 (26.25)	340 (26.48)	337 (26.25)	**370 (28.82)**	**2068 (29.24)**
**Stage**						
	Localized	604 (47.04)	632 (49.22)	604 (47.04)	**553 (43.07)**	**2980 (42.14)**
	Regional	370 (28.82)	373 (29.05)	370 (28.82)	**357 (27.80)**	**1776 (25.11)**
	Distant	159 (12.38)	145 (11.29)	159 (12.38)	**197 (15.34)**	**1175 (16.61)**
	Unknown	151 (11.76)	134 (10.44)	151 (11.76)	**177 (13.79)**	**1141 (16.13)**
**Grade**						
	I	138 (10.75)	168 (13.08)	138 (10.75)	181 (14.10)	**960 (13.57)**
	II	181 (14.10)	198 (15.42)	181 (14.10)	173 (13.47)	**1064 (15.05)**
	III	111 (8.64)	109 (8.49)	111 (8.64)	104 (8.10)	**646 (9.13)**
	IV	15 (1.17)	24 (1.87)	15 (1.17)	17 (1.32)	**77 (1.09)**
	Unknown	839 (65.34)	785 (61.14)	839 (65.34)	809 (63.01)	**4325 (61.16)**
**Surgery type**						
	No surgery	>965 (>75.16)	>965 (>75.16)	**>1038 (>80.84)**	**>1057 (>82.32)**	**5651 (79.91)**
	Tumor destruction	127 (9.89)	127 (9.89)	**109 (8.49)**	**92 (7.17)**	**557 (7.88)**
	Resection	155 (12.07)	155 (12.07)	**93 (7.24)**	**93 (7.24)**	**610 (8.63)**
	Transplant	26 (2.02)	26 (2.02)	**33 (2.57)**	**31 (2.41)**	**157 (2.22)**
	Unknown	<11 (<0.86)	<11 (<0.86)	**<11 (<0.86)**	**<11 (<0.86)**	**97 (1.37)**
**Radiation**						
	No	>1228 (>95.64)	>1228 (>95.64)	>1222 (>95.17)	>1226 (>95.48)	**6588 (93.16)**
	Yes	45 (3.50)	45 (3.50)	51 (3.97)	47 (3.66)	**381 (5.39)**
	Unknown	<11 (<0.86)	<11 (<0.86)	<11 (<0.86)	<11 (<0.86)	**103 (1.45)**
**Chemotherapy**						
	No	921 (71.73)	921 (71.73)	**965 (75.16)**	**985 (76.71)**	**5566 (78.70)**
	Yes	363 (28.27)	363 (28.27)	**319 (24.84)**	**299 (23.29)**	**1506 (21.30)**

**: Suppressed due to sample size ≤10

HCC, hepatocellular carcinoma; SD, standard deviation. Variables controlled in one of the 3 matches but allowed to vary naturally in other matches. The “Asian Patients” column reports the statistical numbers for all Asian patients in the data set. The “Treatment Match” column reports the statistical numbers for the closest non-Hispanic white match, namely the treatment match (which also controls for presentation and demographics variables); the “Presentation Match” column also controls for demographics variables. The “All Whites (Unmatched)” column reports data for all non-Hispanic whites in the data set without matching. Results for each variable that appear to the left of the bold vertical line are for variables included in the match designated by the column. Results to the right of the bold vertical line are for variables not used in the match designated by the column. Percentages or rates bolded imply statistically significant (*P* < 0.05) differences between Asian and non-Hispanic white.

Among all HCC patients, Asians had a lower risk of HCC-specific death compared with NHWs (HR = 0.74, 95% CI: 0.68–0.80). Older age was associated with increased 5-year HCC mortality (HR per 1-year increase in age = 1.01, 95% CI: 1.01–1.01) while more contemporary HCC patients had lower risk of HCC-specific mortality (HR per 1 year beyond 1994 = 0.98, 95% CI: 0.97–0.98). Compared with patients diagnosed with HCC in Western regions, patients diagnosed in Midwestern (HR = 1.09, 95% CI: 1.01–1.18) or Southern (HR = 1.18, 95% CI: 1.10–1.27) regions had a higher risk of HCC-specific mortality. Compared to HCC patients with no comorbid conditions, those with ≥3 comorbidities had only slightly higher risk of HCC-specific mortality (HR = 1.07, 95% CI: 1.00–1.14). For tumor presentation characteristics, more advanced stage at diagnosis and higher tumor grade were associated with increased risk of HCC-specific mortality. Receiving radiation and chemotherapy decreased mortality risk by 29% (HR = 0.71, 95% CI: 0.64–0.79) and 37% (HR = 0.63, 95% CI: 0.59–0.67), respectively. All surgery types decreased mortality risk compared with receiving no surgery at all. The most aggressive surgery procedure such as liver transplantation reduced the risk most by 85% (HR = 0.15, 95% CI: 0.12–0.20) (**Table 2**).

**Table 2:** Hazard ratios and 95% CIs for selected characteristics and HCC 5-year survival among unmatched non-Hispanic whites and Asians (n = 8,356).

Characteristic	Crude HR (95% CI)	Mutually-adjusted HR (95% CI)
Age at diagnosis	1.03 (1.02–1.03)	1.01 (1.01–1.01)
Year of diagnosis	0.96 (0.96–0.97)	0.98 (0.97–0.98)
Gender		
Male	1.00 (ref.)	1.00 (ref.)
Female	1.03 (0.98–1.08)	0.96 (0.91–1.02)
Race		
Non-Hispanic Whites	1.00 (ref.)	1.00 (ref.)
Asians	0.67 (0.62–0.72)	0.74 (0.68–0.80)
Socioeconomic status		
Low	1.00 (ref.)	1.00 (ref.)
High	0.90 (0.86–0.94)	1.00 (0.95–1.05)
Current married		
No	1.00 (ref.)	1.00 (ref.)
Yes	0.83 (0.79–0.87)	0.97 (0.92–1.02)
Living area		
Metropolitan or urban	1.00 (ref.)	1.00 (ref.)
Less urban or rural area	0.84 (0.77–0.91)	0.99 (0.90–1.08)
Region		
West	1.00 (ref.)	1.00 (ref.)
Midwest	1.34 (1.24–1.44)	1.09 (1.01–1.18)
Northeast	1.06 (0.99–1.13)	0.97 (0.90–1.04)
South	1.21 (1.13–1.29)	1.18 (1.10–1.27)
NCI comorbidity index		
0	1.00 (ref.)	1.00 (ref.)
1	0.87 (0.81–0.93)	0.98 (0.92–1.05)
2	0.91 (0.84–0.98)	1.04 (0.97–1.12)
3+	0.90 (0.85–0.97)	1.07 (1.00–1.14)
Stage at diagnosis		
Localized	1.00 (ref.)	1.00 (ref.)
Regional	1.90 (1.79–2.02)	1.62 (1.52–1.72)
Distant	3.18 (2.96–3.42)	2.26 (2.10–2.44)
Unknown	2.35 (2.18–2.52)	1.48 (1.37–1.59)
Tumor Grade		
I	1.00 (ref.)	1.00 (ref.)
II	1.03 (0.94–1.14)	1.28 (1.16–1.41)
III	1.77 (1.59–1.97)	1.67 (1.50–1.86)
IV	1.52 (1.20–1.94)	1.62 (1.27–2.06)
Unknown	1.68 (1.55–1.82)	1.38 (1.27–1.49)
Surgery type		
No surgery	1.00 (ref.)	1.00 (ref.)
Resection	0.24 (0.22–0.27)	0.27 (0.24–0.30)
Liver transplant	0.13 (0.10–0.17)	0.15 (0.12–0.20)
Tumor destruction	0.34 (0.30–0.37)	0.41 (0.37–0.45)
Unknown	0.96 (0.78–1.19)	0.86 (0.68–1.09)
Radiation		
No	1.00 (ref.)	1.00 (ref.)
Yes	0.99 (0.89–1.10)	0.71 (0.64–0.79)
Chemotherapy		
No	1.00 (ref.)	1.00 (ref.)
Yes	0.64 (0.60–0.68)	0.63 (0.59–0.67)

CI, confidence interval; HR, hazard ratio.

**Fig 2:**
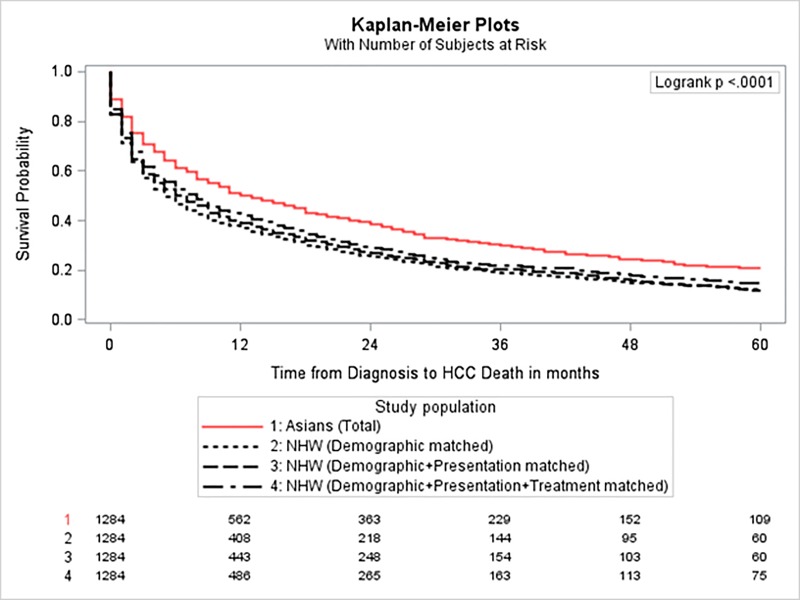
Kaplan-Meier plot for Hepatocellular carcinoma survival for Asian patients and three matched non-Hispanic white populations.

Matching quality by each criteria are shown in **S4 Table**. In each match, the controlled variables had standardized differences < 0.1 SDs, demonstrating successful matches. After 5-years of follow-up, a total of 851 (66.3%) Asians and 5,576 (78.8%) NHWs died due to HCC. Compared with their Asian counterparts, NHW patients with HCC had significantly lower median survival (5 vs. 13 months, P<0.001). Comparing Asians with unmatched (all) NHWs, the absolute difference in 5-year survival was 9.9% (95% CI: 5.7%-14.3%). After accounting for differences in demographic factors (e.g., age, sex, SES,) through the demographics-match, this survival advantage among Asians was reduced to 8.4% (95% CI: 4.6%-12.0%). The disparity remained unchanged however after additionally matching on stage, grade and comorbidities in the presentation match analysis (**Table 3**). Finally, the greatest reduction in the absolute survival difference was achieved through treatment matching. In the final analysis, matching directly on treatment factors, along with propensity score matching of demographics and presentation factors, the absolute difference in 5-year survival was reduced to 5.8% (95% CI: 2.6%-9.3%). The 1- and 2-year survival rates were also consistently higher and risk of HCC-caused morality were consistently lower among Asians than NHWs after all three sequential matchings (**Table 3**). The Kaplan-Meier survival curve also demonstrated a significant lower survival of HCC for all three matched NHW groups compared with Asians (**Fig 2**). The results were unchanged when we matched on or adjusted for the individual conditions used to form the comorbidity index.

**Table 3:** Outcomes of Asian and non-Hispanic white patients with HCC.

Outcome Measure		Asian Patients	Matched non-Hispanic White Patients		
		(n = 1284)	Treatment Match	Presentation Match	Demographics Match
			(n = 1284)	(n = 1284)	(n = 1284)
Survival, median (95%CI), months		13.0 (11.0–15.0)	8.0 (6.0–9.0)	7.0 (5.0–8.0)	5.0 (4.0–6.0)
	*P* value		**< 0.0001**	**< 0.0001**	**< 0.0001**
1-y survival, % (95%CI) ^a^		50.3%	42.0%	39.2%	36.8%
	Survival difference (%) ^b^	NA	8.3% (4.8%-11.3%)	11.1% (7.3%-14.4%)	13.5% (9.3%-16.7%)
	*P* value		**< 0.0001**	**< 0.0001**	**< 0.0001**
	No. of deaths	599	710	737	765
2-y survival, % (95%CI) ^a^		38.5%	28.8%	26.7%	25.4%
	Survival difference (%) ^b^	NA	9.7% (6.8%-13.5%)	11.8% (8.5%-15.5%)	13.1% (10.0%-17.3%)
	*P* value		**< 0.0001**	**< 0.0001**	**< 0.0001**
	No. of deaths	719	846	863	877
5-y survival, % (95%CI) ^a^		20.7%	14.9%	11.8%	12.3%
	Survival difference (%) ^b^	NA	5.8% (2.6%-9.3%)	8.9% (5.4%-12.1%)	8.4% (4.6%-12.0%)
	*P* value		**0.0002**	**< 0.0001**	**< 0.0001**
	No. of deaths	851	947	970	969
Paired Cox model, HR,		NA	0.77 (0.69–0.87)	0.67 (0.59–0.75)	0.67 (0.59–0.75)
Asian: Non-Hispanic White (95%CI)					
	*P* value		**< 0.0001**	**< 0.0001**	**< 0.0001**

^a^ Confidence Intervals calculated by 1000 bootstrap resampling; P-values calculated by using normal distribution of differences of survival rates.

^b^ Survival differences between Asian and matched non-Hispanic white patients.

CI, confidence interval; HR, hazard ratio. Demographics indicates one to one matching of Asian and non-Hispanic white patients on age at diagnosis, sex, year of diagnosis, social economic status and SEER Registry Code; Presentation indicates propensity score matching on demographics variables plus one to one matching on comorbid conditions, stage and grade; Treatment indicates propensity score matching on demographics and presentation plus one to one matching of surgery, chemotherapy, and radiation.

Further stratified analysis by stage at diagnosis showed that that racial disparities of Asians versus NHWs in 5-year survival were attenuated after treatment matching (before treatment matching: 16.1% vs. after treatment matching: 5.6%) among patients with localized stage HCC, but not for regional or distant stage cases (before treatment matching: 8.0% vs. after treatment matching: 7.4%) (**S5 and S6 Tables**). For patients diagnosed prior to 2007, we observed a higher 2-year survival among Asians compared with their NHW counterparts and the difference did not change significantly after treatment matching (before treatment matching: 9.2% vs. after treatment matching: 8.8%). Among patients diagnosed after 2007, we observed an increased 5-year survival for both races especially for Asians (Asians: 40.0% vs. NHWs: 11.9%). Though attenuated after matched on treatment modalities, 5-year survival advantage of Asians over NHWs still remained (before treatment matching: 28.1% vs. after treatment matching: 21.0%) (**S7 and S8 Tables**).

In this large nationwide population-based study, we found a significant lower risk of mortality due to HCC among Asians compared with NHWs. Most of the disparity was due to treatment, whereby Asian patients were more likely than NHW patients to receive curative treatment after HCC diagnosis. The 5-year survival disparity between Asians and NHW patients matched for demographics was 8.4%, or a difference in median survival time of 8 months. Consistent with prior studies, we observed a significant racial disparity in tumor stage and frequency of existing comorbid conditions at HCC diagnosis. However, we found that these factors contributed little to the survival disparity between Asians and NHWs. For NHW patients matched for presentation related factors, the magnitude of the 5-year survival disparity was 8.9%. Compared with NHW patients who both presented and were treated like Asian patients (the treatment match), the disparity in 5-year survival was reduced to 5.8%. As expected, the disparity in treatment had a greater effect when NHW patients were diagnosed with less advanced cancer, similar to the Asian HCC population. In the treatment match analysis among only patients with localized HCC, there was no statistically significant difference in 5-year survival rates.

Our finding of better HCC-specific survival rates among Asian patients compared with NHW patients is consistent with previous studies [5,10,28]. Except for female gender and SEER site, we found that no other demographic factors were associated with HCC mortality. Expectedly, matching on demographic variables therefore only minimally reduced the survival disparity between Asians and NHWs. Stage at diagnosis is an important predictor for HCC prognosis regardless of staging schemes [11,29]. We found in our study a higher fraction of late stage HCCs among NHWs than Asians. To better reflect the comorbidity burden specific for HCC, we incorporated infections of HBV or HCV, as well as histories of NAFLD and AFLD into the “mild liver disease” and “moderate/severe liver disease” categories of the NCI comorbidity index. NAFLD was previously found to be associated with worse HCC prognosis possibly due to later stage at diagnosis [30,31]. In addition, an uprising trend of HCV-caused mortality has been observed in recent decades [17]. A moderate increased risk of HCC-specific mortality was observed in our study cohort with heavier comorbidity burden (NCI comorbidity index≥3). Unlike previous studies [15,16,32], there was no association in our study between marital status, SES and the risk of HCC-specific mortality. Current married and high SES could be a reflection of higher likelihood of diagnosis at earlier stage and receiving treatment such as surgery, radiation or chemotherapy. Nonetheless, the results from our study provide the strongest evidence yet that these (presentation) factors do not explain the observed survival disparity between Asian and NHW HCC patients in the US.

In addition, treatment modalities are important explanatory factors for HCC prognosis. We found that risk of HCC-specific mortality was significantly lower (ranging from 29% to 85%) among patients receiving various surgery types, radiation or chemotherapy compared to patients who did not receive treatment. Compared with NHW patients, we found that Asian patients in the SEER-Medicare database were more likely to receive surgery, including tumor destruction and resection. Importantly, we showed through the treatment match analysis that these differences in receipt of treatment explained a large fraction, but not all, of the survival disparity between Asians and NHWs. However, the contribution of treatment differences to the survival disparity was greatest among patients diagnosed with early stage HCC. In the era of new targeted therapy for advanced HCC such as sorafenib in 2007 [27], we found an even greater survival disparity between Asians and NHWs. Furthermore, this significant survival disparity remained even in the treatment match analysis. It may be that response to treatment (e.g., a biological mechanism), rather than controlling for a disparity in the receipt of any treatment, explains why Asian patients have such a persistent survival advantage over NHW patients.

Our study has several strengths including the population-based sampling design. The SEER-Medicare linked database allowed for a comprehensive assessment and control of comorbidity burdens which are not available in the SEER dataset alone. Also in-patient, out-patient and physicians’ claim data help to better determine and classify the status of receiving various treatment modalities including surgery types, radiation and chemotherapy. Last, the novel minimum distance-based matching strategy enabled us to explain the contributions of various factors sequentially to the observed survival disparity. There are some limitations in our study. First, census-tract level demographic variables such as SES do not convey individual variance within the tract. Second, we could not rule out potential misclassification of treatment status since no chart reviews were conducted to verify treatment coded from Medicare bills. Third, our study population consisted of Medicare beneficiaries aged over 65 years at HCC diagnosis, which may not be generalizable to younger population and other population groups with business insurance or without health insurance coverage. Finally, for all patients, we also saw that a racial disparity in the 5-year survival of 5.8% remained even after matching for presentation and treatment related factors. However, it was anticipated, given that disparities exists between racial/ethnic subgroups in the health care system that some residual survival disparity would remain after accounting for cancer presentation and treatment. For example, bias at the center level for management and treatment of HCC patients may explain some of the residual survival disparity; however, we were unable to examine this in the SEER-Medicare dataset. Likewise, other factors such as diet and genetic predispositions could potentially explain the racial difference in HCC survival, but these are beyond the scope of this study.

## Conclusions

In conclusion, a better 5-year survival of HCC was found among Asians than NHWs for patients diagnosed between 1994 and 2011 in the US. Earlier stage at diagnosis and more likely to receive curative treatments among Asians could partially explain the survival difference between these two racial groups. Future studies are warranted to explore other explanatory factors for the residual racial disparity in HCC survival.

## Supporting information

S1 Table Definition of surgery types. (DOCX)

S2 Table Components of socioeconomic (SES) status and score assignment. (DOCX)

S3 Table Definitions and weights of NCI comorbidity index components. (DOCX)

S4 Table Detailed matching quality by each matching criteria. (DOCX)

S5 Table Outcomes of Asian and non-Hispanic white patients with localized HCC. (DOCX)

S6 Table Outcomes of Asian and non-Hispanic white patients with regional and distant HCC. (DOCX)

S7 Table Outcomes of Asian and non-Hispanic white patients with HCC diagnosis before year 2007. (DOCX)

S8 Table Outcomes of Asian and non-Hispanic white patients with HCC diagnosis after year 2007. (DOCX)
