# Supplementary material for: Factors associated with favorable survival outcomes for Asians with hepatocellular carcinoma: A sequential matching cohort study
Source: PLoS One. 2019 Apr 3;14(4):e0214721. doi: 10.1371/journal.pone.0214721 (PMC6447218; doi:10.1371/journal.pone.0214721)
Supplement: S1 Table — (DOCX) [file pone.0214721.s001.docx]

**Supplemental Table 1. Definition of surgery types**

| **Characteristic** | **Definition** |
| --- | --- |
| No surgery | Site-specific surgery code: 00, 01, 02, 03, 04, 05, 06  OR  Surgery primary site code: 00 |
| Tumor destruction | Site-specific surgery code: 10. 20, 30, 35  OR  Surgery primary site code: 10, 11, 12, 13, 14, 15, 16, 17 |
| Resection | Site-specific surgery code: 40, 50, 60, 68, 80, 90  OR  Surgery primary site code: 20, 21, 22, 23, 24, 25, 26, 30, 36, 37, 38, 39, 50, 51, 52, 59, 60, 61, 65, 75, 80, 90 |
| Liver transplantation | ICD-9-CM code: V42.7, 50.4, 50.5  OR  CPT codes: 47135, 47136, 47140, 47141, 47142, 47143, 47144, 47145, 47146, 47174 |
| Unknown | Site-specific surgery code: 09  OR  Surgery primary site code: 99 |
