# Supplementary material for: Factors associated with favorable survival outcomes for Asians with hepatocellular carcinoma: A sequential matching cohort study
Source: PLoS One. 2019 Apr 3;14(4):e0214721. doi: 10.1371/journal.pone.0214721 (PMC6447218; doi:10.1371/journal.pone.0214721)
Supplement: S2 Table — (DOCX) [file pone.0214721.s002.docx]

**Supplemental Table 2. Components of socioeconomic (SES) status and score assignment**

| **Characteristic** | **Score** |
| --- | --- |
| Median household income, in US dollars |  |
| 1^st^ quartile (≤34,470) | 0 |
| 2^nd^ quartile (>34,470 and ≤46,554) | 1 |
| 3^rd^ quartile (>46,554 and <= 62,747) | 2 |
| 4^th^ quartile (>62,747) | 3 |
| Persons aged 25+ with <12 years of education, in % |  |
| >75% | 0 |
| >50% and ≤75% | 1 |
| >25% and ≤50% | 2 |
| ≤25% | 3 |
| Percent of residents living below poverty, % |  |
| >75% | 0 |
| >50% and ≤75% | 1 |
| >25% and ≤50% | 2 |
| ≤25% | 3 |
