# Supplementary material for: Factors associated with favorable survival outcomes for Asians with hepatocellular carcinoma: A sequential matching cohort study
Source: PLoS One. 2019 Apr 3;14(4):e0214721. doi: 10.1371/journal.pone.0214721 (PMC6447218; doi:10.1371/journal.pone.0214721)
Supplement: S3 Table — (DOCX) [file pone.0214721.s003.docx]

**Supplemental Table 3. Definitions and weights of NCI comorbidity index components**

| **Condition** | **Definition** | **Weight** |
| --- | --- | --- |
| Acute Myocardial Infarction | ICD-9 Diagnosis: 410.xx with inpatient length of stay >2 days | 1 |
| History of Myocardial Infarction | ICD-9 Diagnosis: 412.bb | 1 |
| Congestive Heart Failure | ICD-9 Diagnosis: 398.91, 425.4x-425.5x, 425.7x-425.9x, 428.xx | 1 |
| Peripheral Vascular Disease | ICD-9 Diagnosis: 093.0x, 440.xx-441.xx, 442.0x-442.8x, 443.1x-443.9x, 447.70-447.73, 785.4x, V43.4x | 1 |
|  | ICD-9 Procedure: 00.60, 38.13, 38.14, 38.15, 38.16, 38.18, 38.33, 38.34, 38.36, 38.38, 38.43, 38.44, 38.46, 38.48, 38.68, 39.25, 39.29 |  |
| Cerebrovascular Disease (CVD) | ICD-9 Diagnosis: 430.xx- 438.xx | 1 |
|  | ICD-9 Procedure: 00.61, 00.62, 00.63, 00.65, 38.12, 38.32, 38.42, 39.22, 39.28, 39.74 |  |
| Chronic Obstructive Pulmonary Disease (COPD) | ICD-9 Diagnosis: 416.8x-416.9x, 490.xx-496.xx, 500.xx-505.xx, 506.4x, 519.1x | 1 |
| Dementia | ICD-9 Diagnosis: 290.xx, 291.0x-291.2x, 292.82, 294.1x, 331.0x-331.2x, 331.82 | 1 |
| Paralysis (Hemiplegia or Paraplegia) | ICD-9 Diagnosis: 342.xx, 344.0x-344.6x, 344.9x | 2 |
| Diabetes | ICD-9 Diagnosis: 250.bb, 250.0x-250.3x | 1 |
| Diabetes with Complications | ICD-9 Diagnosis: 250.4x-250.9x, 362.0x | 2 |
| Renal Disease | ICD-9 Diagnosis: 403.01, 403.11, 403.91, 404.02, 404.03, 404.12, 404.13, 404.92, 404.93, 582.xx-583.xx, 585.xx-586.xx, 588.xx, V42.0x, V45.1x, V56.xx | 2 |
|  | ICD-9 Procedure: 39.27, 39.42, 39.95, 54.98, 55.69 |  |
| Mild Liver Disease | ICD-9 Diagnosis: 070.32-070.33, 070.54, 070.70, 571.0x-571.6x, 571.8x, 571.9x, V02.61, V02.62 | 1 |
| Moderate/Severe Liver Disease | ICD-9 Diagnosis: 070.22-070.23, 070.41, 070.44, 070.71, 456.0x-456.2x, 572.2x-572.8x, V42.7x | 3 |
|  | ICD-9 Procedure: 39.1b, 42.91, 50.5x |  |
| Peptic Ulcer Disease | ICD-9 Diagnosis: 531.xx-534.xx | 1 |
| Rheumatologic Disease | ICD-9 Diagnosis: 710.0x, 710.1x, 710.4x, 714.0x-714.2x, 714.81, 725.bb | 1 |
| AIDS | ICD-9 Diagnosis: 042.xx-044.x, V08.bb, 795.71 | 6 |
