# Supplementary material for: Factors associated with favorable survival outcomes for Asians with hepatocellular carcinoma: A sequential matching cohort study
Source: PLoS One. 2019 Apr 3;14(4):e0214721. doi: 10.1371/journal.pone.0214721 (PMC6447218; doi:10.1371/journal.pone.0214721)
Supplement: S4 Table — (DOCX) [file pone.0214721.s004.docx]

**Supplemental Table 4. Detailed matching quality by each matching criteria**

| **Variable** | |  | **Non-Hispanic White Patients, n (%)** | | | | | | | |
| --- | --- | --- | --- | --- | --- | --- | --- | --- | --- | --- |
|  |  | **Asian Patients** | **Treatment Match** | | | **Presentation Match** | | **Demographic Match** | | **All Whites (Unmatched)** |
|  |  | **(n = 1284)** | **(n = 1284)** | | **SDD** | **(n = 1284)** | **SDD** | **(n = 1284)** | **SDD** | **(n = 7072)** |
| **Mean age at diagnosis (SD), y** | | 75.40 (6.19) | 75.15 (6.59) | | 0.03 | 75.45 (6.64) | -0.01 | 75.41 (6.39) | 0.00 | 75.71 (6.45) |
| **Mean diagnosis year (SD)** | | 2004 (4.39) | 2004 (4.72) | | 0.00 | 2004 (4.70) | 0.00 | 2004 (4.77) | 0.00 | 2004 (4.66) |
| **Female** | | 451 (35.12) | 443 (34.50) | | 0.01 | 426 (33.18) | 0.04 | 451 (35.12) | 0.00 | **2223 (31.43)** |
| **SES** | |  |  | |  |  |  |  |  |  |
|  | **High** | >632 (>49.22) | | >671 (>52.26) | -0.06 | >646 (>50.31) | -0.02 | >632 (>49.22) | 0.00 | 3392 (47.96) |
|  | **Low** | 641 (49.92) | | 602 (46.88) | 0.06 | 627 (48.83) | 0.02 | 641 (49.92) | 0.00 | 3667 (51.85) |
|  | **Unknown** | <11 (<0.86) | | <11 (<0.86) | 0.00 | <11 (<0.86) | 0.02 | <11 (<0.86) | 0.00 | 13 (0.19) |
| **SEER registry site** | |  |  | |  |  |  |  |  |  |
| 1 | San Francisco | 201 (15.65) | 206 (16.04) | | -0.02 | 196 (15.26) | 0.02 | 201 (15.65) | 0.00 | **288 (4.07)** |
| 2 | Connecticut | 16 (1.25) | 12 (0.93) | | 0.01 | 16 (1.25) | 0.00 | 16 (1.25) | 0.00 | **635 (8.98)** |
| 20 | Detroit | 18 (1.40) | 14 (1.09) | | 0.01 | 19 (1.48) | 0.00 | 18 (1.40) | 0.00 | **626 (8.85)** |
| 21 | Hawaii | 39 (3.04) | 36 (2.80) | | 0.02 | 32 (2.49) | 0.07 | 39 (3.04) | 0.00 | **39 (0.55)** |
| 22 | Iowa | ** | ** | | -0.01 | ** | 0.00 | ** | 0.00 | **486 (6.87)** |
| 23 | New Mexico | ** | ** | | 0.01 | ** | -0.01 | ** | 0.00 | **140 (1.98)** |
| 25 | Seattle | 98 (7.63) | 106 (8.26) | | -0.02 | 96 (7.48) | 0.01 | 98 (7.63) | 0.00 | **519 (7.34)** |
| 26 | Utah | ** | ** | | 0.01 | ** | -0.01 | ** | 0.00 | **175 (2.47)** |
| 27 | Atlanta | 13 (1.01) | 12 (0.93) | | 0.00 | 13 (1.01) | 0.00 | 13 (1.01) | 0.00 | **197 (2.79)** |
| 31 | San Jose | 148 (11.53) | 126 (9.81) | | 0.11 | 119 (9.27) | 0.16 | 148 (11.53) | 0.00 | **153 (2.16)** |
| 35 | Los Angeles | 380 (29.60) | 344 (26.79) | | 0.11 | 344 (26.79) | 0.11 | 380 (29.60) | 0.00 | **486 (6.87)** |
| 37 | Rural Georgia | ** | ** | | 0.00 | ** | 0.00 | ** | 0.00 | **22 (0.31)** |
| 41 | Greater California | 303 (23.60) | 350 (27.26) | | -0.10 | 369 (28.74) | -0.15 | 303 (23.60) | 0.00 | **984 (13.91)** |
| 42 | Kentucky | ** | ** | | 0.00 | ** | 0.00 | ** | 0.00 | **468 (6.66)** |
| 43 | Louisiana | ** | 12 (0.93) | | -0.04 | ** | 0.00 | ** | 0.00 | **419 (5.92)** |
| 44 | New Jersey | 37 (2.88) | 40 (3.12) | | -0.01 | 46 (3.58) | -0.02 | 37 (2.88) | 0.00 | **930 (13.15)** |
| 47 | Greater Georgia | ** | ** | | 0.00 | ** | 0.01 | ** | 0.00 | **505 (7.14)** |
| **NCI Comorbidity Index** | |  |  | |  |  |  |  |  |  |
|  | 0 | 285 (22.20) | 331 (25.78) | | -0.05 | 285 (22.20) | 0.00 | **357 (27.80)** | **-0.13** | **1876 (26.53)** |
|  | 1 | 407 (31.70) | 382 (29.75) | | 0.05 | 407 (31.70) | 0.00 | **332 (25.86)** | **0.13** | **1854 (26.22)** |
|  | 2 | 255 (19.85) | 231 (17.99) | | 0.03 | 255 (19.85) | 0.00 | **225 (17.52)** | **0.06** | **1274 (18.01)** |
|  | >=3 | 337 (26.25) | 340 (26.48) | | -0.01 | 337 (26.25) | 0.00 | **370 (28.82)** | **-0.06** | **2068 (29.24)** |
| **Stage** | |  |  | |  |  |  |  |  |  |
|  | Localized | 604 (47.04) | 632 (49.22) | | -0.04 | 604 (47.04) | 0.00 | **553 (43.07)** | **0.08** | **2980 (42.14)** |
|  | Regional | 370 (28.82) | 373 (29.05) | | -0.01 | 370 (28.82) | 0.00 | **357 (27.80)** | **0.02** | **1776 (25.11)** |
|  | Distant | 159 (12.38) | 145 (11.29) | | 0.03 | 159 (12.38) | 0.00 | **197 (15.34)** | **-0.08** | **1175 (16.61)** |
|  | Unknown | 151 (11.76) | 134 (10.44) | | 0.04 | 151 (11.76) | 0.00 | **177 (13.79)** | **-0.06** | **1141 (16.13)** |
| **Grade** | |  |  | |  |  |  |  |  |  |
|  | I | 138 (10.75) | 168 (13.08) | | -0.07 | 138 (10.75) | 0.00 | 181 (14.10) | -0.10 | **960 (13.57)** |
|  | II | 181 (14.10) | 198 (15.42) | | -0.04 | 181 (14.10) | 0.00 | 173 (13.47) | 0.02 | **1064 (15.05)** |
|  | III | 111 (8.64) | 109 (8.49) | | 0.01 | 111 (8.64) | 0.00 | 104 (8.10) | 0.02 | **646 (9.13)** |
|  | IV | 15 (1.17) | 24 (1.87) | | -0.07 | 15 (1.17) | 0.00 | 17 (1.32) | -0.01 | **77 (1.09)** |
|  | Unknown | 839 (65.34) | 785 (61.14) | | 0.09 | 839 (65.34) | 0.00 | 809 (63.01) | 0.05 | **4325 (61.16)** |
| **Surgery type** | |  |  | |  |  |  |  |  |  |
|  | No surgery | >965 (>75.16) | | >965 (>75.16) | 0.00 | **>1038 (>80.84)** | **-0.14** | **>1057 (>82.32)** | **-0.17** | **5651 (79.91)** |
|  | Tumor destruction | 127 (9.89) | 127 (9.89) | | 0.00 | **109 (8.49)** | **0.05** | **92 (7.17)** | **0.10** | **557 (7.88)** |
|  | Resection | 155 (12.07) | 155 (12.07) | | 0.00 | **93 (7.24)** | **0.17** | **93 (7.24)** | **0.17** | **610 (8.63)** |
|  | Transplant | 26 (2.02) | 26 (2.02) | | 0.00 | **33 (2.57)** | **-0.04** | **31 (2.41)** | **-0.03** | **157 (2.22)** |
|  | Unknown | <11 (<0.86) | <11 (<0.86) | | 0.00 | **<11 (<0.86)** | **0.01** | **<11 (<0.86)** | **-0.01** | **97 (1.37)** |
| **Radiation** |  |  |  | |  |  |  |  |  |  |
|  | No | >1228 (>95.64) | >1228 (>95.64) | | 0.00 | >1222 (>95.17) | 0.02 | >1226 (>95.48) | 0.01 | **6588 (93.16)** |
|  | Yes | 45 (3.50) | 45 (3.50) | | 0.00 | 51 (3.97) | -0.02 | 47 (3.66) | -0.01 | **381 (5.39)** |
|  | Unknown | <11 (<0.86) | <11 (<0.86) | | 0.00 | <11 (<0.86) | -0.01 | <11 (<0.86) | 0.00 | **103 (1.45)** |
| **Chemotherapy** | |  |  | |  |  |  |  |  |  |
|  | No | 921 (71.73) | 921 (71.73) | | 0.00 | **965 (75.16)** | **-0.08** | **985 (76.71)** | **-0.12** | **5566 (78.70)** |
|  | Yes | 363 (28.27) | 363 (28.27) | | 0.00 | **319 (24.84)** | **0.08** | **299 (23.29)** | **0.12** | **1506 (21.30)** |

HCC, hepatocellular carcinoma; SD, standard deviation; SDD, standard deviation difference.

**: Suppressed due to sample size ≤10

Variables controlled in one of the 3 matches but allowed to vary naturally in other matches. The “Asian Patients” column reports the statistical numbers for all Asian patients in the data set. The “Treatment Match” column reports the statistical numbers for the closest non-Hispanic white match, namely the treatment match (which also controls for presentation and demographics variables); the “Presentation Match” column also controls for demographics variables. The “All Whites (Unmatched)” column reports data for all non-Hispanic whites in the data set without matching. Results for each variable that appear to the left of the bold vertical line are for variables included in the match designated by the column. Results to the right of the bold vertical line are for variables not used in the match designated by the column. Percentages or rates bolded imply statistically significant (*P* < 0.05) differences between Asian and non-Hispanic white.
