# Supplementary material for: Factors associated with favorable survival outcomes for Asians with hepatocellular carcinoma: A sequential matching cohort study
Source: PLoS One. 2019 Apr 3;14(4):e0214721. doi: 10.1371/journal.pone.0214721 (PMC6447218; doi:10.1371/journal.pone.0214721)
Supplement: S5 Table — (DOCX) [file pone.0214721.s005.docx]

**Supplemental Table 5. Outcomes of Asian and non-Hispanic white patients with localized HCC**

| **Outcome Measure** | | **Asian Patients** | **Matched non-Hispanic White Patients** | |
| --- | --- | --- | --- | --- |
|  |  | **(n = 342)** | **Treatment Match** | **Presentation Match** |
|  |  |  | **(n = 342)** | **(n = 342)** |
| Survival, median (95%CI), months | | 34.0 (27.0-43.0) | 29.0 (24.0-38.0) | 20.0 (15.0-23.0) |
|  | *P* value |  | **0.204** | **< 0.0001** |
| 1-y survival, % (95%CI) ^a^ | | 73.2% | 70.5% | 62.2% |
|  | Survival difference (%) ^b^ | NA | 2.7% (-4.1%, 9.5%) | 11.0% (3.9%, 18.1%) |
|  | *P* value |  | **0.442** | **0.0027** |
|  | No. of deaths | 88 | 98 | 121 |
| 2-y survival, % (95%CI) ^a^ | | 58.1% | 55.0% | 41.4% |
|  | Survival difference (%) ^b^ | NA | 3.1% (-4.7%, 10.9%) | 16.7% (8.8%, 24.6%) |
|  | *P* value |  | **0.439** | **< 0.0001** |
|  | No. of deaths | 132 | 142 | 178 |
| 5-y survival, % (95%CI) ^a^ | | 37.6% | 32.0% | 21.5% |
|  | Survival difference (%) ^b^ | NA | 5.6% (-3.0%, 14.2%) | 16.1% (7.9%, 24.3%) |
|  | *P* value |  | **0.202** | **< 0.0001** |
|  | No. of deaths | 178 | 185 | 217 |
| Paired Cox model, HR, | | NA | 0.88 (0.67-1.15) | 0.60 (0.46-0.78) |
| Asian: Non-Hispanic White (95%CI) | |  |  |  |
|  | *P* value |  | **0.341** | **0.0001** |
