# Supplementary material for: Factors associated with favorable survival outcomes for Asians with hepatocellular carcinoma: A sequential matching cohort study
Source: PLoS One. 2019 Apr 3;14(4):e0214721. doi: 10.1371/journal.pone.0214721 (PMC6447218; doi:10.1371/journal.pone.0214721)
Supplement: S6 Table — (DOCX) [file pone.0214721.s006.docx]

**Supplemental Table 6. Outcomes of Asian and non-Hispanic white patients with regional and distant HCC**

| **Outcome Measure** | | **Asian Patients** | **Matched non-Hispanic White Patients** | |
| --- | --- | --- | --- | --- |
|  |  | **(n = 253)** | **Treatment Match** | **Presentation Match** |
|  |  |  | **(n = 253)** | **(n = 253)** |
| Survival, median (95%CI), months | | 6.0 (4.0-8.0) | 3.0 (3.0-4.0) | 3.0 (2.0-4.0) |
|  | *P* value |  | **0.0047** | **0.0004** |
| 1-y survival, % (95%CI) ^a^ | | 33.8% | 25.4% | 22.4% |
|  | Survival difference (%) ^b^ | NA | 8.4% (0.2%, 16.6%) | 11.4% (3.3%, 19.5%) |
|  | *P* value |  | **0.045** | **0.006** |
|  | No. of deaths | 158 | 181 | 188 |
| 2-y survival, % (95%CI) ^a^ | | 21.9% | 13.0% | 12.9% |
|  | Survival difference (%) ^b^ | NA | 8.9% (1.8%, 16.0%) | 9.0% (1.9%, 16.1%) |
|  | *P* value |  | **0.014** | **0.013** |
|  | No. of deaths | 181 | 208 | 208 |
| 5-y survival, % (95%CI) ^a^ | | 11.8% | 4.4% | 3.8% |
|  | Survival difference (%) ^b^ | NA | 7.4% (0.8%, 14.0%) | 8.0% (1.6%, 14.4%) |
|  | *P* value |  | **0.028** | **0.016** |
|  | No. of deaths | 193 | 220 | 222 |
| Paired Cox model, HR, | | NA | 0.79 (0.61-1.04) | 0.75 (0.58-0.97) |
| Asian: Non-Hispanic White (95%CI) | |  |  |  |
|  | *P* value |  | **0.0635** | **0.0265** |
