# Supplementary material for: Factors associated with favorable survival outcomes for Asians with hepatocellular carcinoma: A sequential matching cohort study
Source: PLoS One. 2019 Apr 3;14(4):e0214721. doi: 10.1371/journal.pone.0214721 (PMC6447218; doi:10.1371/journal.pone.0214721)
Supplement: S7 Table — (DOCX) [file pone.0214721.s007.docx]

**Supplemental Table 7. Outcomes of Asian and non-Hispanic white patients with HCC diagnosis before year 2007**

| **Outcome Measure** | | **Asian Patients** | **Matched non-Hispanic White Patients** | |
| --- | --- | --- | --- | --- |
|  |  | **(n = 259)** | **Treatment Match** | **Presentation Match** |
|  |  |  | **(n = 259)** | **(n = 259)** |
| Survival, median (95%CI), months | | 8.0 (6.0-11.0) | 4.0 (3.0-6.0) | 5.0 (3.0-8.0) |
|  | *P* value |  | **0.0189** | **0.0410** |
| 1-y survival, % (95%CI) ^a^ | | 39.6% | 31.1% | 32.8% |
|  | Survival difference (%) ^b^ | NA | 8.5% (-0.1%, 17.1%) | 6.8% (-1.8%, 15.4%) |
|  | *P* value |  | **0.0530** | **0.1235** |
|  | No. of deaths | 144 | 171 | 166 |
| 2-y survival, % (95%CI) ^a^ | | 29.8% | 20.6% | 21.0% |
|  | Survival difference (%) ^b^ | NA | 9.2% (1.2%, 17.2%) | 8.8% (0.8%, 16.8%) |
|  | *P* value |  | **0.0240** | **0.0317** |
|  | No. of deaths | 164 | 194 | 192 |
| 5-y survival, % (95%CI) ^a^ | | 11.0% | 9.4% | 7.9% |
|  | Survival difference (%) ^b^ | NA | 1.6% (-4.7%, 7.9%) | 3.1% (-3.1%, 9.3%) |
|  | *P* value |  | **0.6190** | **0.3305** |
|  | No. of deaths | 199 | 216 | 216 |
| Paired Cox model, HR, | | NA | 0.72 (0.56-0.93) | 0.77 (0.60-1.00) |
| Asian: Non-Hispanic White (95%CI) | |  |  |  |
|  | *P* value |  | **0.0108** | **0.0515** |
