# Supplementary material for: Factors associated with favorable survival outcomes for Asians with hepatocellular carcinoma: A sequential matching cohort study
Source: PLoS One. 2019 Apr 3;14(4):e0214721. doi: 10.1371/journal.pone.0214721 (PMC6447218; doi:10.1371/journal.pone.0214721)
Supplement: S8 Table — (DOCX) [file pone.0214721.s008.docx]

**Supplemental Table 8. Outcomes of Asian and non-Hispanic white patients with HCC diagnosis after year 2007**

| **Outcome Measure** | | **Asian Patients** | **Matched non-Hispanic White Patients** | |
| --- | --- | --- | --- | --- |
|  |  | **(n = 108)** | **Treatment Match** | **Presentation Match** |
|  |  |  | **(n = 108)** | **(n = 108)** |
| Survival, median (95%CI), months | | 27.0 (16.0-60.0) | 13.0 (7.0-22.0) | 12.0 (7.0-23.0) |
|  | *P* value |  | **0.0246** | **0.0050** |
| 1-y survival, % (95%CI) ^a^ | | 62.3% | 50.1% | 48.7% |
|  | Survival difference (%) ^b^ | NA | 12.2% (-1.5%, 25.9%) | 13.6% (-0.1%, 27.3%) |
|  | *P* value |  | **0.0818** | **0.0516** |
|  | No. of deaths | 39 | 49 | 51 |
| 2-y survival, % (95%CI) ^a^ | | 51.4% | 37.3% | 37.0% |
|  | Survival difference (%) ^b^ | NA | 14.1% (-0.4%, 28.6%) | 14.4% (-0.1%, 28.9%) |
|  | *P* value |  | **0.0574** | **0.0515** |
|  | No. of deaths | 48 | 60 | 60 |
| 5-y survival, % (95%CI) ^a^ | | 40.0% | 19.0% | 11.9% |
|  | Survival difference (%) ^b^ | NA | 21.0% (3.2%, 38.8%) | 28.1% (10.6%, 45.6%) |
|  | *P* value |  | **0.0210** | **0.0016** |
|  | No. of deaths | 52 | 67 | 70 |
| Paired Cox model, HR, | | NA | 0.84 (0.53-1.35) | 0.70 (0.44-1.11) |
| Asian: Non-Hispanic White (95%CI) | |  |  |  |
|  | *P* value |  | **0.4738** | **0.1302** |
